# Investigating the Moderating Effect of Attitudes Toward One’s Own Aging on the Association Between Body Mass Index and Executive Function in Older Adults

**DOI:** 10.3390/geriatrics10040105

**Published:** 2025-08-06

**Authors:** Akihiko Iwahara, Taketoshi Hatta, Reiko Nakayama, Takashi Miyawaki, Seiji Sakate, Junko Hatta, Takeshi Hatta

**Affiliations:** 1 Faculty of Psychology and Collaboration, Kyoto Women’s University, Kyoto 6058501, Japan; hattata@kyoto-wu.ac.jp; 2 Faculty of Home Economics, Kyoto Women’s University, Kyoto 6058501, Japan; nakayamr@kyoto-wu.ac.jp (R.N.); miyawakt@kyoto-wu.ac.jp (T.M.); sakate@kyoto-wu.ac.jp (S.S.); 3 Faculty of Psychology, Aichi Gakuin University, Nisshin 4700195, Japan; hatta105@dpc.agu.ac.jp; 4 Graduate School of Environmental Studies, Nagoya University, Nagoya 4648601, Japan; hatta@tamateyama.ac.jp

**Keywords:** body mass index, obesity, cognitive function, executive function, attitudes toward own aging

## Abstract

**Background:** This cross-sectional study examined the association between body mass index (BMI) and executive function (EF) in older adults, with a focus on the moderating role of attitudes toward own aging (ATOA). **Method:** A total of 431 community-dwelling elderly individuals from Yakumo Town and Kyoto City, Japan, participated between 2023 and 2024. EF was assessed using the Digit Cancellation Test (D-CAT), and ATOA was measured via a validated subscale of the Philadelphia Geriatric Center Morale Scale. **Results:** Multiple linear regression analyses adjusted for demographic and health covariates revealed a significant interaction between BMI and ATOA in the younger-old cohort. Specifically, higher BMI was associated with lower executive function only in individuals with lower ATOA scores. No such association was observed in those with more positive views on aging. **Conclusions:** These results indicate that positive psychological constructs, particularly favorable self-perceptions of aging, may serve as protective factors against the detrimental cognitive consequences of increased body mass index in younger-old populations.

## 1. Introduction

The prevalence of obesity/overweight is rising problematically in developed and developing nations worldwide [1]. The World Health Organization reported that 2.5 billion adults aged 18 years and older were overweight, including over 890 million adults who were living with obesity in 2022 [2]. Among older adults, obesity has been shown to negatively impact various health outcomes, including physical function, quality of life, cognitive decline, and increased mortality risk [3]. In addition to its clinical implications, obesity/overweight contributes significantly to the global economic burden by increasing healthcare expenditures and is associated with more than 3.7 million deaths annually [2].

While it is clear that obesity/overweight correlates with poor mental and physical health, recent studies have linked obesity and overweight to poorer cognitive functioning [4]. Several studies have reported that obesity/overweight is associated with a decline in overall cognitive abilities [5,6,7], including specific domains such as memory, attention, language, and executive function (EF). Among the various cognitive domains, executive function—a higher-order set of neurocognitive processes responsible for goal-directed behavior, inhibitory control, cognitive flexibility, and decision-making—has been identified as particularly vulnerable to the adverse effects of obesity. EF refers to a set of higher-order cognitive processes that allow one to plan, organize, and successfully execute purposeful, goal-directed, and future-oriented actions [8]. The construct of EF refers to a set of higher-order cognitive processes that, while interrelated, are functionally and anatomically dissociable. This conceptualization is well captured by the widely recognized “unity and diversity” model of EF [9]. According to this framework, EF comprises three core components: (1) inhibition—the capacity to suppress prepotent or inappropriate responses (for example, as measured by the Stroop Color–Word Test); (2) shifting—the ability to switch attention between tasks or mental representations (for example, as measured by the Trail Making Test Part B); and (3) updating—the ability to monitor and revise information held in working memory (n-back task and the Digital Cancellation Test). Although these components do not encompass the full range of EFs, they represent principal domains that have been consistently examined across diverse neuropsychological and experimental paradigms. Recent systematic reviews have further emphasized the link between obesity and deficits in EF, proposing that impairments in EF may contribute to maladaptive eating behaviors through diminished inhibitory control [4,6,7]. Compared to individuals with healthy weight, those with obesity exhibit reduced resistance to interference and are more prone to making impulsive dietary choices. These findings underscore the role of EF in dietary decision-making and physical activity regulation, suggesting that EF may serve as a critical cognitive mechanism underlying the maintenance of healthy weight.

Although obesity and overweight have been theoretically linked to impairments in EF, empirical findings in the literature remain inconclusive. As mentioned above, several studies have reported significant deficits in executive functioning among individuals with obesity compared to those with normal weight. In contrast, other investigations have failed to observe statistically significant differences in executive performance between obese and normal-weight individuals [10]. These inconsistencies may be attributable to variations in study design, sample characteristics, assessment tools, or the specific components of EF examined. Such inconsistencies across studies may be attributable to a range of methodological and conceptual factors, including heterogeneity in participant age ranges, the presence of comorbid conditions (e.g., depression, diabetes), variations in body mass index (BMI) classification criteria, and differences in the sensitivity and specificity of the neuropsychological tasks used to assess EF. Additionally, the multidimensional nature of EF itself may contribute to these discrepancies. For instance, specific components such as inhibitory control and cognitive flexibility may be more vulnerable to obesity-related alterations than others.

A comprehensive meta-analysis conducted by Yang et al. [4] sought to elucidate contextual factors that may moderate the relationship between overweight status and EF performance. The authors systematically examined the potential moderating effects of various variables, including age and sex and the specific EF measures employed across studies. However, age and sex were not identified as significant moderators in the relationship between obesity and EF in their analysis. This finding suggests that the association between obesity/overweight and executive dysfunction may be independent of these demographic variables. As the authors proposed, the lack of consistent findings across studies may be attributable to unmeasured or inadequately controlled confounding factors. Despite the examination of several potential moderators, most did not demonstrate statistically significant effects. The authors noted that methodological inconsistencies—particularly the heterogeneity of EF tasks used across studies—may have obscured potential moderating effects. Furthermore, they highlighted the possibility that other relevant factors influencing this association may not have been captured in the analysis. To address these limitations, the authors recommended that future research control for additional variables, such as participants’ clinical status, educational attainment, socioeconomic background, use of weight-related medications, and the presence of psychiatric comorbidities.

The present study aims to examine whether psychological factors—specifically, self-perceptions of aging (SPA)—may underlie the inconsistent associations observed between BMI and EF. Prior research has demonstrated that positive self-perceptions of aging can exert a beneficial influence on a range of health outcomes, including improved functional health over time, enhanced survival rates, and greater health-related psychological resources such as perceived control and will to live [11,12]. In a large-scale longitudinal study utilizing data from 5702 older adults in the nationally representative U.S. Health and Retirement Study, Levy et al. [13] found that individuals with more positive self-perceptions of aging were significantly less likely to become obese over a six-year period. Specifically, those with the highest self-perceptions of aging scores exhibited a 27% lower risk of obesity compared to individuals with average self-perceptions of aging scores. Further evidence from a 12-year longitudinal study involving a representative sample of 500 older adults in Germany indicated that self-perceptions of aging were positively associated with the maintenance of fluid intelligence, although no significant relationship was observed with crystallized intelligence [14]. In a subsequent study, Siebert et al. [15] reported that more negative self-perceptions of aging significantly predicted a steeper decline in cognitive functioning among the older cohort, even after controlling for sociodemographic factors, baseline health status, and depressive symptoms. Building on these findings, the present study hypothesizes that self-perceptions of aging may serve as a moderating factor in the relationship between BMI and EF in older adults.

## 2. Method

### 2.1. Participants and Ethical Considerations

#### 2.1.1. Participants

The participants were from the Yakumo study database and the Higashiyama study database. Just a brief note for the Yakumo Study and the Higashiyama Study.

The Yakumo study is an ongoing cohort study that began in 1981 as part of a joint project between Yakumo Town in Hokkaido and the Nagoya University Graduate School of Medicine. The participants were community dwellers from a typical rural Japanese population who had a history of employment in agriculture, fishing, forestry, or office work. The research teams consisted of various fields, such as epidemiology, internal medicine, orthopedics, urology, ophthalmology, otolaryngology, and neuropsychology. In the Yakumo Study, annual health examinations are conducted each August among community residents. Because participation is voluntary, the frequency of participation varies among individuals—some attend every year, while others participate once every two to three years. Consequently, data collection occurs either annually or at intermittent intervals depending on the participant. This study was newly designed to examine the association between SPA and cognitive as well as physical functions. A new questionnaire survey related to SPA was conducted in the above-mentioned Yakumo study field. Present data were from 484 participants aged 65 years or older at the time of participation, who took part in the neuropsychology team examination from 2023 to 2024. Community dwellers participated voluntarily and managed their daily lives by themselves. Some participants participated annually, whereas others participated every 2 or 3 years. In cases of overlapping participation, data from the year in which the participants performed in 2023 were used.

The Higashiyama study was initiated in 2023 as a joint project between Kyoto Women’s University and the Higashiyama Ward of Kyoto City. Present data was from 132 participants who were residents aged 65 years or older living in Higashiyama ward. The Higashiyama ward is adjacent to the Shimogyo, Nakagyo, and Kamigyo wards that make up the central part of Kyoto City, and the target population was residents of an area that meets the definition of an urban area that constitutes a busy, commercial district such as Gion. Therefore, the participants in this study can be considered elderly adults with different background cultures, with the population consisting of a typical local municipality and residents of a typical large city in Japan.

Of the initial 622 participants recruited from both regions, individuals with missing data on key variables were excluded from the analysis. As a result, the final analytical sample comprised 431 participants.

#### 2.1.2. Ethical Considerations

This study was conducted in accordance with the ethical standards outlined in the Declaration of Helsinki and was approved by the Scientific Committee of Kyoto Women’s University (2023-40) and Nagoya University (2011#643). Prior to participation, written informed consent was obtained from all individuals after providing a full explanation of the study’s aims and procedures. Strict measures were taken to ensure the confidentiality and security of personal data and research findings, in compliance with relevant institutional and international ethical guidelines.

### 2.2. Measures and Statistical Analysis

#### 2.2.1. Predictor

BMI was assess ed using standardized anthropometric measurements of weight and height. Weight was measured to the nearest 0.1 kg using a calibrated digital scale, and height was measured to the nearest 0.1 cm using a standard stadiometer. All anthropometric assessments were conducted in accordance with standardized procedures recommended by the World Health Organization [16]. BMI was calculated as weight in kilograms divided by the square of height in meters (kg/m^2^).

#### 2.2.2. Outcome

The Digital Cancellation Test (D-CAT) was utilized as an indicator of EF. The D-CAT is a paper-and-pencil-type screening test for attention and EF that basically follows Sohlberg and Matter’s attention mode [17]. The D-CAT sheet comprised 12 rows of 50 digits. Each row contained 5 sets of numbers from 0 to 9 arranged randomly. In the D-CAT, participants were instructed to search for the target number and delete each number with a slash mark as quickly and accurately as possible. There were two conditions in the D-CAT: the target was a single digit (D-CAT1), and the target was three digits (D-CAT3); the former addressed mainly information processing speed and focusing, while the latter addressed information processing speed, focusing, and working memory assessment (particularly the updating component of EF). The reliability and validity of the D-CAT have been reported elsewhere [18].

#### 2.2.3. Moderator and Covariates

For our aging self-perceptions measure, we used the ‘‘Attitudes toward Own Aging (ATOA)’’ subscale that Liang and Bollen [19] based on five items from the Philadelphia Geriatric Center Morale Scale [20]. This subscale consists of the following items: (1) ‘‘Things keep getting worse as I get older’’, (2) ‘‘I have as much pep as I did last year’’, (3) ‘‘As you get older, you are less useful’’, (4) ‘‘I am as happy now as I was when I was younger’’, and (5) ‘‘As I get older, things are (better, worse, or the same) as I thought they would be’’. In this study, following previous studies [21], the total score of the four items excluding the fifth item was used as the SPA score. The covariates included in all models were selected because they have been found to be related to the predictor, the outcome, or both. The covariates consisted of age, sex, education, depression (K6) [22], systolic blood pressure (SBP), diastolic blood pressure (DBP), and hemoglobin A1c (HbA1c). The Kessler Psychological Distress Scale (K6) is a validated, standardized self-report instrument designed to assess global non-specific psychological distress. It comprises six items that evaluate the frequency of symptoms related to depression and anxiety over the past 30 days, providing a brief yet effective screening measure for population-level mental health surveillance. Based on scores from K6, participants were classified into two categories: those with suspected depression (K6 score ≥ 5) and those without depression (K6 score < 5).

#### 2.2.4. Data Analysis

Univariate analyses were conducted to describe baseline demographic characteristics for the total study sample and stratified by SPA groups. The SPA variable was dichotomized at the sample mean, resulting in two groups: negative and positive SPA. Independent samples *t*-tests and χ^2^ tests were used to examine group differences in demographic and clinical variables.

**Figure 1:**
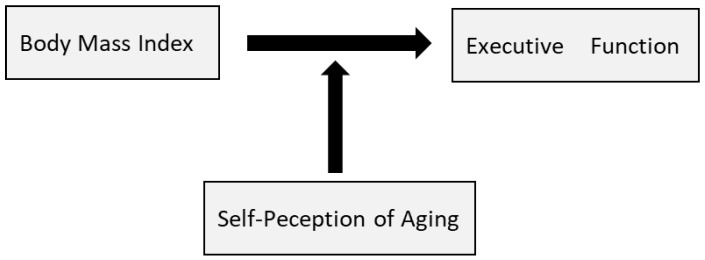
The hypothesis of this study: moderating effect of self-perception of aging on the association between body mass index and executive function.

To investigate the main hypothesis, the association between BMI and EF, as assessed by the Digit Cancellation Test (D-CAT), was examined with SPA as a moderator (see Figure 1). Multivariate linear regression analyses were employed to assess the effects of the predictor variables on D-CAT performance. In the regression model, BMI served as the primary independent variable, and D-CAT performance was the dependent variable. Covariates included age, sex, educational attainment, depression, SBP, DBP, and hemoglobin A1c (HbA1c). Moderation analysis was conducted by including an interaction term (BMI × SPA) in the regression model.

Additionally, given the potential for differential effects of self-perceptions of aging on the relationship between BMI and EF across age groups, participants were stratified into two categories based on chronological age. Individuals aged 65 to 74 years were classified as the younger-old adults group, while those aged 75 years and older were classified as the older-old adults group. To further explore the moderating role of self-perceptions of aging (SPA) in the relationship between BMI and EF across different age groups, stratified analyses were conducted separately for younger-old adults and older-old adults cohorts. These stratified analyses aimed to determine whether the interaction between BMI and SPA on EF varies by age group, thereby capturing potential age-related differences in the psychological and physiological mechanisms underlying this relationship.

All regression coefficients were estimated using the method of least squares. Statistical significance was determined using a two-tailed alpha level of 0.05. All statistical analyses were performed using IBM SPSS Statistics for Windows, Version 30.0 [23] and HAD Version 18.0 [24].

## 3. Results

### 3.1. Characteristics of the Participants

The demographic and clinical characteristics of the participants, stratified by SPA, are presented in Table 1. The overall sample had a mean age of 73.72 years (SD = 5.99), and 62.98% were female. Among younger-old adults, the mean age was 69.84 years (SD = 2.95), with 59.23% female participants. In older-old adults, the mean age was 80.37 years (SD = 4.60), and 68.59% were female. No significant differences were observed in age or sex distribution between the positive and negative SPA groups within either younger-old adults or older-old adults. With regard to educational attainment, the overall mean was 12.72 (SD = 2.28) years, with younger-old adults averaging 13.27 (SD = 2.14) years and older-old adults averaging 12.11 (2.21) years of education.

The mean score on the ATOA scale was 2.94 (SD = 1.14) for the overall sample, with scores of 3.05 (SD = 1.11) and 2.83 (SD = 1.17) for younger-old adults and older-old adults, respectively. Not surprisingly, there was a significant difference in ATOA scores between the positive SPA group and the negative SPA group (younger-old adults: *t* (231) = 26.29, *p* < 0.001; older-old adults: *t* (154) = 21.48, *p* < 0.001). A total of 27 participants (6.94%) were classified as experiencing depressive symptoms based on K6 scores, with 14 individuals (6.01%) in younger-old adults and 13 individuals (8.33%) in older-old adults meeting the criteria for suspected depression. In both age groups, the prevalence of depressive symptoms was lower among individuals with positive SPA compared to those with negative SPA (younger-old adults: *χ^2^* (1) = 12.51, *p* < 0.001; older-old adults: *χ^2^* (1) = 16.35, *p* < 0.001).

The mean BMI of participants in the present sample was 23.42 (SD = 3.37) overall, 23.44 (SD = 3.38) among younger-old adults, and 23.11 (SD = 3.26) among older-old adults. Similarly, the mean SBP was 135.67 mmHg (SD = 19.42) overall, 133.72 mmHg (SD = 18.49) in younger-old adults, and 139.28 mmHg (SD = 19.86) in older-old adults. DBP averaged 73.98 mmHg (SD = 11.70) overall, 76.16 mmHg (SD = 11.88) in younger-old adults, and 72.74 mmHg (SD = 40.70) in older-old adults. HbA1c levels were 5.92% (SD = 0.56) in the total sample, 5.90% (SD = 0.51) among younger-old adults, and 5.92% (SD = 0.53) among older-old adults.

### 3.2. A Moderating Effect of SPA on the Association Between BMI and EF

Table 2 presents the results of the multiple linear regression analyses conducted separately for younger-old adults and older-old adults, with D-CAT3 performance as the outcome variable. The primary independent variable was BMI, with attitude toward own aging (ATOA) included as a moderator. Covariates included age, sex, years of education, depressive symptoms, SBP, DBP, and HbA1c.

**Figure 2:**
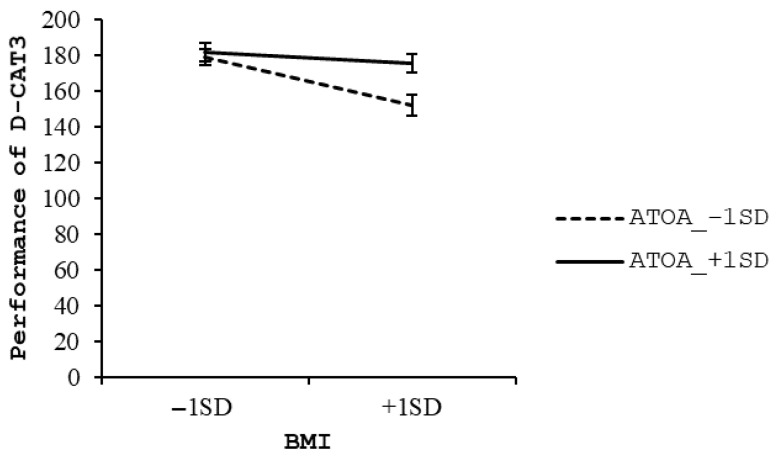
Simple slope analysis in younger-old adults group.

In younger-old adults, the regression model was statistically significant (*F* (10, 206) = 5.99, *p* < 0.001), accounting for 18.8% of the variance in D-CAT3 performance (*R*^2^ = 0.188). BMI was a significant negative predictor of D-CAT3 scores (*β* = −0.20, *t* = −3.02, *p* = 0.003), and the interaction term between BMI and ATOA was also statistically significant (*β* = 0.13, *t* = 2.07, *p* = 0.015), suggesting that ATOA moderated the association between BMI and EF. Among the covariates, age (*β* = −0.22, *p* < 0.001), sex (*β* = 0.15, *p* = 0.027), and education (*β* = 0.26, *p* = 0.003) were also significant predictors. Simple slope analysis revealed that lower performance on the D-CAT3 was associated with higher BMI in the lower ATOA group (*β* = −0.33, *t* = −3.47, *p* = 0.001), whereas no such association was observed in the higher ATOA group (*β* = −0.08, *t* = −0.89, *p* = 0.373) (see Figure 2).

In contrast, in older-old adults, the regression model was also significant (*F* (10, 126) = 2.41, *p* = 0.012), but it explained a smaller proportion of the variance in D-CAT3 scores (*R*^2^ = 0.094). Neither BMI (*β* = −0.13, *t* = −1.43, *p* = 0.155) nor the interaction term (BMI × ATOA; *β* = 0.002, *t* = 0.02, *p* = 0.985) were statistically significant, indicating no moderating effect of ATOA in this subgroup. Among the covariates, only age was a significant predictor of D-CAT3 performance (*β* = −0.24, *p* = 0.006).

## 4. Discussion

The present study provides novel evidence suggesting that SPA may moderate the association between BMI and EF in older adults. This finding aligns with prior research demonstrating impaired EF in individuals with overweight or obesity [4]. To our knowledge, this is the first study to investigate the moderating role of SPA in the relationship between BMI and EF. Furthermore, we stratified participants into two age groups—younger-old adults and older-old adults—to explore whether the interaction between BMI and SPA differs by age, thereby capturing potential age-related variations in the psychological and physiological mechanisms underlying this association. Stratified analyses revealed that among individuals with high SPA, no significant correlation was observed between BMI and EF. In contrast, among individuals with low SPA, higher BMI was associated with poorer EF. These interactions were observed in younger-old adults, but not in older-old adults. These results support the hypothesis that higher BMI is linked to diminished executive functioning in older adults. However, the absence of a significant association in those with high SPA may help explain inconsistencies observed in the previous literature regarding the relationship between BMI and EF.

Before interpreting the results, it is important to describe the characteristics of the study participants, as these factors may influence the interpretation and generalizability of the findings. The characteristics of the present sample were generally consistent with those reported in previous population-based studies conducted in Japan, although some distinctions were observed. While the sample included a relatively higher proportion of women, the sex distribution remained comparable to prior Japanese studies [21,25]. Educational attainment among participants appeared relatively high, suggesting that the sample may comprise individuals with greater health consciousness. ATOA (Attitudes Toward One’s Own Aging) scores were comparable to, though slightly higher than, those reported by Nakagawa and Yasumoto (2019) [21], indicating a generally positive outlook on aging. Furthermore, given that the estimated prevalence of depression among older adults in Japan is approximately 10%, the low levels of depressive symptoms observed in the present sample suggest a relatively favorable mental health profile. Nevertheless, despite high SPA (self-perceptions of aging) scores and low depressive symptoms, cognitive performance as assessed by the D-CAT was within normative ranges reported in previous studies [18], with no significant group differences observed across SPA levels. These findings suggest that while psychological well-being was relatively high, executive functioning did not surpass age-expected levels, indicating that the influence of positive SPA on cognitive performance may be complex. Clinical health indicators were also consistent with national averages, including those reported in the Hisayama Study [25], suggesting that the physical health status of the sample was broadly normative. No significant differences in these physical health parameters were observed between SPA groups in either the younger-old or older-old subgroups.

### 4.1. Theoretical Interpretation of the Relationship Between BMI and EF

Obesity-related inflammation has been proposed as a key biological mechanism linking elevated BMI to impaired EF. Obesity is associated with chronic low-grade systemic inflammation, and accumulating evidence suggests that this sustained inflammatory state may contribute to cognitive decline, particularly in domains related to executive functioning. Recent studies have indicated that obesity-induced activation of the innate immune system leads to persistent inflammatory responses, which may in turn negatively impact EF [26,27,28]. This relationship is further supported by the immunologic model of self-regulatory failure, which posits that increased inflammatory activity—especially involving components of the immune system responsible for inflammation—can impair executive functioning by disrupting self-regulatory processes [29].

Moreover, it has been suggested that the association between BMI and EF may be bidirectional. That is, poor executive functioning may also serve as a risk factor for weight gain, as impaired cognitive control can lead to maladaptive health behaviors [30]. The dual-process model of behavior provides a theoretical framework for this bidirectional association. According to this model, behavior is shaped by the interaction between an impulsive system, which responds automatically to environmental cues, and an executive control system, which enables self-regulation [31]. In individuals with elevated BMI, a heightened automatic approach tendency toward high-calorie foods, combined with reduced executive control, may increase susceptibility to obesity-promoting behaviors such as excessive caloric intake and poor dietary choices. Conversely, individuals with stronger EF may be better equipped to resist these impulses and maintain healthier behaviors, thereby mitigating the risk of obesity [4].

The association between elevated BMI and impaired EF is supported by neuroimaging studies demonstrating structural and functional abnormalities in the brains of individuals with obesity. Specifically, altered connectivity has been observed among key neural circuits involved in cognitive control, including the fronto-striato-parietal network [32]. Functional magnetic resonance imaging (fMRI) studies have reported obesity-related hypoactivation in prefrontal cortical regions, such as the inferior frontal gyrus, during the performance of EF tasks [33,34]. Additionally, reduced functional connectivity within the fronto-striatal circuitry has been documented during the processing of food-related cues in individuals with obesity. Moreover, decreased centrality in brain regions such as the left medial frontal gyrus and lateral occipital cortex has been reported among obese individuals [32]. The medial frontal gyrus is a critical region involved in multiple neural pathways responsible for attention regulation, executive functioning, and motor control. In older adults, obesity substantially increases the risk of developing type 2 diabetes, a condition known to induce structural brain changes and elevate the risk of neurodegenerative disorders.

In recent years, several systematic reviews have examined the effects of physical activity on EF in obese and overweight populations [35,36]. These reviews consistently report that exercise interventions yield significant cognitive benefits. Guo et al. [35] concluded that structured exercise interventions, including aerobic and resistance training, are associated with significant improvements in EF among obese older adults. These findings suggest that physical activity may serve as a non-pharmacological strategy to mitigate cognitive decline in this population. Regular aerobic exercise or resistance training over a period of six months has been demonstrated to significantly improve neurocognitive function, including executive processes, in older adults with obesity. In addition to cognitive benefits, resistance training has been associated with improved metabolic outcomes in patients with type 2 diabetes, such as enhanced insulin sensitivity and better glycemic control. Moreover, interventions aimed at reducing sedentary behavior—for instance, incorporating 30 min walking sessions following prolonged periods of sitting—have also been shown to effectively enhance EF in this population. Taken together, these findings suggest that lower BMI may facilitate better EF via complex interactions among biological, neurophysiological, and behavioral pathways.

### 4.2. No Significant Association Between BMI and EF Among Older Adults with a High Level of SPA

In later life, a positive SPA has been identified as a significant predictor of healthier aging trajectories and increased longevity, whereas a negative SPA is associated with reduced life expectancy and diminished functional health outcomes [37]. Levy et al. [13] showed that individuals with more positive SPA are significantly less likely to become obese over a six-year follow-up period compared to those with negative SPA. The promotion of positive societal and self-directed attitudes toward aging—recognizing older adults as competent and aging itself as a phase marked by potential—can encourage older individuals to adopt resilient, proactive behaviors that align with these expectations. Empirical evidence suggests that SPA may influence engagement in health-promoting behaviors. For instance, individuals with more positive SPA are more likely to participate in regular physical activity [38].

These findings underscore the role of SPA in modulating health behavior patterns across the aging process. Attitudes toward health behavior change also appear to differ as a function of SPA. Older adults with a more positive view on aging not only demonstrate greater functional optimism but also tend to maintain future-oriented goals and aspirations. These goals—such as travel, hobbies, or caregiving—require the maintenance of good health, thereby reinforcing the motivation to engage in behaviors conducive to well-being [38]. Furthermore, individuals with higher SPA often report a greater sense of purpose in life, which is typically characterized by goal-directed activity and engagement in personally meaningful experiences [39]. A stronger sense of purpose has been associated with increased participation in health-promoting behaviors such as physical activity and social engagement, which may offer protective effects against risk factors like depression, physical inactivity, and obesity [40]. Taken together, these findings suggest that older adults with a positive SPA are more likely to engage in behaviors that support physical and cognitive health, possibly due to their optimistic outlook and enhanced sense of purpose. This psychological resilience may attenuate the negative impact of elevated BMI on EF and broader cognitive decline. Our findings emphasize the importance of considering positive psychosocial factors such as SPA when examining the relationship between physiological indicators such as BMI and cognitive outcomes in aging populations.

### 4.3. Dissociation Between BMI and EF in the Late Elderly

The association between BMI and dementia risk appears to vary with age [41]. While midlife obesity is generally associated with increased risk of Alzheimer’s disease (AD), low BMI in late life has also been linked to elevated dementia risk [42,43]. Notably, a U-shaped relationship has been observed in individuals under 76 years of age, with both underweight and obese individuals showing higher risk, whereas in those aged 76 and older, higher BMI was associated with lower risk [44]. The “protected survivor model” offers a potential explanation for these age-related shifts. It suggests that as individuals age, those more vulnerable to the adverse effects of certain risk factors (e.g., obesity) may not survive, leaving a subset of “protected” individuals in whom these factors appear less harmful or even protective [45]. These findings suggest that, among the older-old adults, BMI was not independently associated with EF, nor did SPA significantly moderate this relationship.

### 4.4. Additional Psychosocial Considerations and Cultural Context of SPA

In addition to depressive symptoms, other psychosocial factors such as perceived stress, anxiety, and social support have also been shown to influence the relationship between BMI and cognitive function in older adults [46,47]. For example, higher stress and anxiety levels are associated with poorer executive functioning, whereas stronger perceived social support may serve as a protective factor. Although this study statistically controlled for depressive symptoms using the K6 scale, future research should examine a broader range of psychological variables to better understand their potential confounding or moderating effects on the association between BMI and EF.

Moreover, SPA are strongly influenced by cultural values, norms, and societal attitudes toward aging [48]. In collectivist societies like Japan, where respect for elders and interdependence are emphasized, SPA may differ substantially from those in Western, individualistic cultures. Given that the current data were drawn exclusively from a Japanese sample, the generalizability of these findings to other cultural contexts may be limited. Future cross-cultural studies are warranted to clarify how SPA interacts with health outcomes across diverse populations.

### 4.5. Limitations

Several limitations of the present study should be acknowledged. First, only the D-CAT was used to assess EF. While the D-CAT is a valid and efficient screening tool, it primarily measures aspects of attention and processing speed. It does not comprehensively assess other key components of EF, such as working memory, cognitive flexibility, or inhibitory control. Future studies should incorporate a broader range of validated neuropsychological instruments—such as the Stroop Color–Word Test (for inhibition), the Trail Making Test Part B (for task-switching), or the Wisconsin Card Sorting Test (for cognitive flexibility)—to provide a more comprehensive evaluation of EF domains.

Second, the study did not collect data on important lifestyle-related variables that may influence both BMI and EF. For instance, dietary quality (e.g., adherence to a Mediterranean diet) and levels of physical activity (e.g., daily step counts or moderate-to-vigorous exercise minutes per week) are well-established predictors of cognitive function. The absence of such data limits our ability to control for potential confounding effects and to explore mediating mechanisms between BMI and EF.

Third, the cross-sectional design precludes any causal inferences regarding the directionality of the association between BMI and EF. Longitudinal research is necessary to determine whether higher BMI leads to impaired EF, whether poor EF contributes to unhealthy behaviors and weight gain, or whether these relationships are bidirectional.

Fourth, the sample consisted primarily of relatively healthy, well-educated, and psychologically resilient older adults, many of whom demonstrated high levels of SPA and low levels of depressive symptoms. This homogeneity likely reflects a selection bias, as participants who volunteer for cognitive aging studies often have greater health consciousness and functional capacity. As such, the findings may not generalize to more diverse populations, such as those with lower socioeconomic status, chronic health conditions, or limited access to health-promoting resources. Future research should aim to include more representative samples, including middle-aged individuals and those with varying levels of cognitive, physical, and psychosocial functioning, in order to enhance the external validity and translational potential of the results.

## 5. Conclusions

This study examined whether SPA moderates the relationship between BMI and EF in older adults. In younger-old adults, a moderating effect of SPA on the association between BMI and EF was observed. Among individuals with low SPA, this relationship may be attributed to biological and neurological mechanisms through which obesity negatively affects executive processes. Conversely, in those with high SPA, positive psychological characteristics may promote health-enhancing behaviors, thereby buffering the cognitive impact of elevated BMI. In older-old adults, the absence of significant associations among BMI, SPA, and EF may reflect the “protected survivor model,” which posits that individuals who live to advanced age may represent a resilient subgroup. These findings highlight the importance of considering psychosocial moderators such as SPA when investigating the link between physiological risk factors and cognitive function in aging populations.

## Figures and Tables

**Table 1:** Sample characteristics.

Variables	Overall (*n* = 389)	Younger-Old Adults					Older-Old Adults			
		All (*n* = 233)	Positive SPA (*n* = 174)	Negative SPA (*n* = 59)	*p*-Value		all (*n* = 156)	Positive SPA (*n* = 103)	Negative SPA (*n* = 53)	*p*-Value
age	73.72 (5.99)	69.84 (2.95)	69.91 (2.88)	69.64 (3.16)	0.502		80.37 (4.60)	80.19 (4.66)	80.72 (4.53)	0.254
sex, female (%)	245 (62.98)	138 (59.23)	101 (56.7)	27 (45.8)	0.143		107 (68.59)	71 (65.1)	36 (60.0)	0.507
education	12.72 (2.28)	13.27 (2.14)	13.19 (2.11)	13.49 (2.24)	0.348		12.11 (2.21)	12.23 (2.19)	11.78 (2.19)	0.207
ATOA	2.94 (1.14)	3.05 (1.11)	3.59 (0.49)	1.44 (0.68)	**<0.001**		2.83 (1.17)	3.55 (0.50)	1.43 (0.77)	**<0.001**
D-CAT1	262.49 (67.73)	283.91 (70.27)	287.70 (65.81)	272.73 (81.63)	0.204		243.80 (52.31)	250.69 (47.72)	228.36 (57.88)	**0.017**
D-CAT3	161.59 (39.22)	173.06 (39.03)	175.66 (36.41)	165.41 (45.38)	0.11		151.71 (33.39)	154.71 (31.16)	145.89 (36.97)	0.097
depression	27 (6.94)	14 (6.01)	5 (2.87)	9 (15.25)	**<0.001**		13 (8.33)	2 (1.94)	11 (20.75)	**<0.001**
BMI (kg/m^2^)	23.42 (3.37)	23.44 (3.38)	23.63 (3.47)	22.89 (3.04)	0.072		23.11 (3.26)	23.15 (3.17)	23.02 (3.44)	0.921
SBP (mmHg)	135.67 (19.42)	133.72 (18.49)	133.57 (19.00)	134.17 (17.07)	0.421		139.28 (19.86)	139.85 (20.40)	138.17 (18.90)	0.572
DBP (mmHg)	73.98 (11.70)	76.16 (11.88)	77.07 (12.19)	73.48 (10.56)	**0.041**		72.74 (10.70)	72.81 (10.69)	72.62 (10.80)	0.644
HbA1c (%)	5.92 (0.56)	5.90 (0.51)	5.89 (0.47)	5.92 (0.62)	0.745		5.92 (0.53)	5.89 (0.54)	6.00 (0.51)	0.345

Values are presented as means (SD) or *n* (%). *p* values were calculated using *t*-test for continuous variables and χ2 test for categorical variables. Missing values are not displayed in this table. SPA: self-perception of aging, ATOA: attitudes toward own agin, D-CAT: digit cancelation test, BMI: body mass index, DBP: diastolic blood pressure, SBP: systolic blood pressure, HbA1c: hemoglobin A1c.

**Table 2:** Multiple regression analysis in the younger-old adults group and the older-old adults group.

	Younger-Old Adults			Older-Old Adults	
	*β* (SE)	*p*-Value		*β* (SE)	*p*-Value
age	−0.19 (0.84)	**0.003**		−0.24 (0.64)	**0.006**
sex	0.15 (5.11)	**0.027**		0.0.07 (2.75)	0.433
education	0.26 (1.71)	**<0.001**		0.14 (2.27)	0.107
depression	−0.09 (11.23)	0.162		−0.18 (11.18)	0.054
SBP (mmHg)	0.06 (0.18)	0.508		−0.05 (0.17)	0.612
DBP (mmHg)	−0.00 (0.28)	0.957		0.09 (0.32)	0.398
HbA1c (%)	−0.05 (4.85)	0.472		0.10 (5.94)	0.295
BMI (kg/m^2^)	−0.20 (0.78)	**0.003**		−0.13 (0.92)	0.155
ATOA	0.17 (2.45)	**0.015**		0.02 (2.56)	0.850
BMI*ATOA	0.13 (0.65)	**0.040**		0.00 (0.63)	0.985
R-Square	0.23			0.16	
Adjust R-Square	0.19			0.09	

## Data Availability

The data that support the findings of this study are not publicly available due to participant confidentiality and institutional policy but may be available from the corresponding author upon reasonable request and with appropriate ethical approval.

## Abbreviations

The following abbreviations are used in this manuscript:

BMI	Body Mass Index
EF	Executive Function
SPA	Self-Perception of Aging
ATOA	Attitudes Toward Own Aging
D-CAT	Digit Cancellation Test
SBP	Systolic Blood Pressure
DBP	Diastolic Blood Pressure
